# Does fear of infection affect people’s dental attendance during COVID-19? A Chinese example to examine the association between COVID anxiety and dental anxiety

**DOI:** 10.3389/froh.2023.1236387

**Published:** 2023-10-09

**Authors:** Siyang Yuan, Yuanna Zheng, Zhe Sun, Gerry Humphris

**Affiliations:** ^1^ Dental Health Services Research Unit, School of Dentistry, University of Dundee, Dundee, United Kingdom; ^2^ School/Hospital of Stomatology, Zhejiang Chinese Medical University, Hangzhou, China; ^3^ School of Medicine, University of St Andrews, St Andrews, United Kingdom

**Keywords:** dental anxiety, fear of infection, COVID-19, dental visit, dental care, patient

## Abstract

**Introduction:**

Little is known about the psychological and behavioural effect of COVID-19 pandemic on patients and their reaction to dental visiting. Patients may delay attendance due to fears of contracting the corona virus at the dentist. The study aims to confirm the psychometric properties of the two COVID-19 scales and then use dental patient responses to assess the associations between the COVID-19 Anxiety Scale (CAS), dental anxiety (MDAS) and the clinical care COVID-19 Anxiety (CCAS).

**Methods:**

A cross-sectional study was conducted among 503 patients visiting a polyclinic of a stomatological hospital in East China in 2020. Patients completed a survey consisting of demographical information, dental attendance, COVID-19 Anxiety Scale, Clinical Care COVID-19 Anxiety Scale and Modified Dental Anxiety Scale. Confirmatory factor analysis was conducted to determine the psychometric characteristics. A further structural model was tested with the combined measurement model and a path analysis was calculated.

**Results:**

The sample (*n* = 503) consisted of more women than men (63% vs. 37%). A fifth of the sample (21%) claimed regular dental attendance and just over a third (35%) reported delaying their dental visit due to the pandemic. Our analysis showed that both CAS and CCAS possessed a unidimensional structure. The MDAS was divided into anticipatory and treatment components as separate latent variables. The anticipatory component (MDAS_A) had some association to CCAS through its expression on treatment dental anxiety (MDAS_T). General anxiety about COVID (CAS) had a direct effect on CCAS. The fit statistics were acceptable [Chi-square = 183.27, df = 68, *p* < .001; CFI = 0.973; RMSEA = 0.058 (95%CIs: 0.048–0.068)] and the Standardised Root Mean Square Residuals (SRMR) index was 0.041.

**Discussion:**

The Clinical Care COVID-19 Anxiety has shown satisfactory psychometric properties. Both dental anxiety and general anxiety about the pandemic have strong associations to patients’ fear of contracting corona virus when using dental facilities. Our study has practical implications to help healthcare providers better understand how environmental stressors influence patients’ overall concerns on infection risks and appropriate dental treatments during the pandemic.

## Introduction

The COVID-19 pandemic has raised concerns among the public and health providers. The consequences of contracting the disease are varied, ranging from mild to catastrophic personal effects. Patients with vulnerabilities such as chronic respiratory conditions or other long-term medical conditions are especially susceptible (1). The consequences of contracting COVID-19 can be substantial and life changing, not only from the increased risk of early death but also the chronic effects of what is termed ‘long COVID’ (2). Other complications are evident such as cardiovascular diseases, acute liver and kidney injuries as well as neurological conditions (3). Health services were requested to compromise certain forms of treatments to maintain health professional safety as well as for the patient. This was illustrated within dentistry during COVID pandemic by the limitation of various dental treatments that require aerosol-related treatments such as use of the high-speed rotor drill to reduce the risk of COVID-19 transmission (4).

The reported high mortality and morbidity of COVID-19, in social media and the press, may deter patients from seeking timely dental care. Preventive measures such as social distancing, mask wearing as well as restrictions on aerosol-generating procedures that are relied upon heavily in dentistry may exacerbate the situation of reduced utilisation of dental health service. Whilst dental anxiety has been well reported for its strong association with patients’ avoidance of using routine dental care (5), little is known about the psychological and behavioural effect of COVID-19 pandemic on patients and their reaction to dental visiting. Patients may delay attendance due to fears of contracting the corona virus at the dentist. They may simply prefer to wait before they visit a dentist as they believe they will not receive optimum treatment (6).

During the COVID-19 pandemic, it is likely that dentally anxious patients will experience increased anxiety related to their perceived risk of infection, uncertainty about the pandemic and the fast-evolving changes to the healthcare systems. The Transactional Model of Stress and Coping Theory (7) suggests that people may respond to the perceived stressor in different ways, depending on their individual characteristics and contextual factors. For the COVID-19 pandemic, patients who see the pandemic as a significant threat to their health may experience higher level of anxiety, compared to those who perceive the threat as normal or lower. In addition, patients who have a history of anxiety (e.g., dental anxiety) may be more susceptible to COVID related anxiety. It would be predicted that more intense interventions such as active dental treatments (e.g., a scale and polish) would amplify the level of anxiety experienced by the patient. Hence a further prediction that appears logical is that patients who are dentally anxious are more likely to consider dental treatment as a greater threat to contracting COVID-19 than those who are relaxed about exposure to the dental surgery environment.

**Figure 1:**
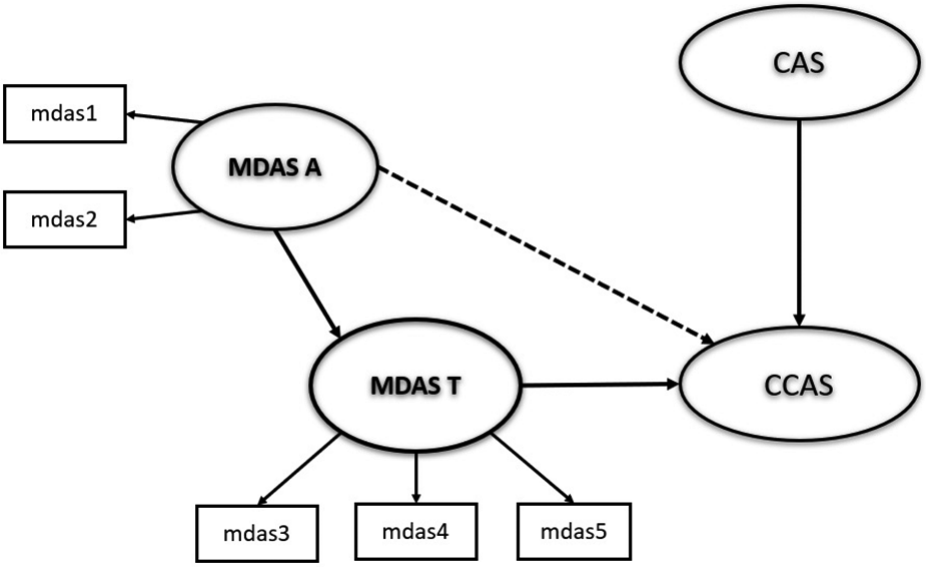
Hypothetical model to describe inter-relationships between dental anxiety (modified dental anxiety scale-anticipatory: MDAS-A and treatment: MDAS-T components) and clinical COVID anxiety (clinical COVID anxiety scale: CCAS) and COVID anxiety (COVID anxiety scale: CAS). Convention of ellipses to denote latent variables.

Based on the Transactional Model of Stress and Coping (7), we therefore present a simple structural model to summarise the network of key relationships that describe the prediction of clinical care COVID-19 anxiety (Figure 1). In this model, the primary appraisal refers to patients’ appraisal of threatening situations which indicate their own dental anxiety (assessed by MDAS) and their perceived risk of COVID-19 infection (assessed using the general COVID-19 Anxiety Scale). Such perception of threats will be determined in a secondary appraisal of patients’ own ability to cope with such stressful situation, which is linked closely to patient’s anxiety about receiving dental care during the pandemic. Hence, two distinct paths aid our understanding of patient’s development of anxiety associated with receiving dental care. The first is based upon the fear of COVID-19 infection itself whereas the second pathway is via the general apprehensiveness of a dental visit and then more strongly by the threat from certain types of invasive treatment itself (e.g., local anaesthetic injection). It is acknowledged that there may be some overlap in a general sense of anxiety that features in both the broader apprehensiveness of dental visiting itself and fear of COVID-19 infection. This is reflected in the covariation indicated by the curved double arrow.

Hence, this study aims to confirm the psychometric properties of the two recent COVID-19 scales and then use patient responses to assess the associations between the COVID-19 Anxiety Scale (CAS), dental anxiety (MDAS) and the Clinical care COVID-19 Anxiety (CCAS) in a dental hospital sample of patients in East China. The specific objectives are:

1. To establish the psychometric properties of the Clinical care COVID-19 Anxiety Scale in a dental attending sample in a dental hospital in East China.
2. To investigate the role of dental anxiety (anticipatory and treatment aspects) and COVID-19 anxiety in predicting anxiety about contracting COVID-19 from dental attendance and not receiving appropriate dental care.

## Method

### Sample and setting

A cross-sectional study was conducted among patients visiting a polyclinic of the Stomatological Hospital affiliated to Zhejiang Chinese Medical University in Hangzhou, China between August and October 2020.

We estimated using the “power r squared” procedure in STATA (8) that with q covariates entered into a linear regression model we would require a sample minimum of 386 respondents to detect a r square value of 5% of the variance of the dependent construct CCAS with alpha set to the conventional 0.05 at 90% power.

Hangzhou, as the capital city of Zhejiang Province, has nearly 12 million in 2020 with an average age of 38.77 years. The proportion of residents aged between 16 and 59 years stood at 70% (9). A non-probability convenience sample of adult patients was used. All patients who visited the polyclinic during the study period were invited to take part. The purpose of the study including the Patient Information Sheet was discussed in the waiting area of the hospital.

The inclusion criteria were those aged 18 years or older and attended the polyclinic for either a dental examination or a dental treatment. Patients were excluded if they were:

a. Unable to read or write,
b. Under 18 years of age,
c. For follow up treatment for a programme already planned with the hospital service.

### Procedures

After completing the written consent form of the study, the patient was invited to complete the self-administered survey questionnaire in the waiting room and asked to return the survey sheet to the reception prior to entry into the clinic. Survey sheets were collected and appended for collection. The anonymised data were transferred to electronic media and sent securely by email spreadsheet attachment to SY in the UK for data processing and analysis.

### Questionnaire

The questionnaire is comprised of the following sections.

[1] Demographic information

The first part of the questionnaire included patient’s age, sex and educational background.

[2] Dental attendance

Patients were asked for their previous experience of attending a dentist, their perceived dental needs as well as the delay of attending dental services. These items included: (i) the usual reason to attend dental appointments (for ‘regular dental examination’ or ‘ to fix dental problems/others’), (ii) their reason to see the dentist today to identify if this was for a dental examination or for treatment that addresses their perceived treatment needs, (iii) whether their dental attendance was delayed due to COVID-19, and to what extent (‘none’, ‘a little’ or ‘a great deal’).

[3] Coronavirus Anxiety Scale (CAS)

The CAS (10) is a 5-item scale that was developed at start of the COVID-19 pandemic to evaluate the frequencies of patient’s anxious symptoms including feeling dizzy, sleep disturbance, tonic immobility, eating disorder and somatic distress caused by the anxiety of COVID-19. Each item is rated on a 5-point Likert scale to measure the frequencies of these anxious symptoms ranging from 1 “not at all” to 5 “nearly every day”.

The literature shows the CAS is found to possess good reliability and some evidence of validity (10); however, this measure only focuses on the physiological symptoms of anxiety that are known to be individually specific. It would be predicted, we believe, that those patients with high levels of anxiety are more likely to report greater concerns and ultimately anxiety about the clinical aspects of receiving dental treatment with the risk of COVID-19 infection. This is, however, not easily interpreted from using the CAS. Hence, to investigate this possibility the current authors designed a short 4-item measure to assess the patients’ anxiety about contracting the virus when visiting a clinical service. The development of CCAS can allow us to further our understanding in how these anxiety stimulating factors (i.e., anxieties about dental treatments and the pandemic) may influence patients’ overall anxiety about risk of COVID-19 infection from visiting a dental service.

[4] Clinical care COVID-19 Anxiety Scale (CCAS)

The CCAS is a 4-item scale developed to test the patients’ anxiety about contracting the corona virus when visiting a clinical service and whether they feel that the treatment they receive is optimum or being compromised. It directly asks the patient’s anxiety about receiving timely and effective treatment as well as their fears of contracting and surviving from COVID-19 when visiting a clinical service. The responses range from 1 “not at all” to 5 “all the time”. This measure has shown previously good reliability (Cronbach’s alpha = 0.34) and construct validity with general anxiety and lack of association with depression (11), e.g., predicted positive relation.

[5] Modified Dental Anxiety Scale (MDAS)

MDAS (12) contains five items to assess the patient’s anticipatory and treatment anxiety using a 5-Likert point scale (1 = not anxious, 5 = extremely anxious). The five items were summed with the total score ranging from 5 to 25. The cut-off score is 19 which indicates the patient with a score of 19 or more is regarded as highly dentally anxious. The reliability of the MDAS is acceptable with proven validity (13).

### Statistical analysis

Statistical analyses were performed using STATA version 15 (8). Alpha was set to 0.05 throughout. The psychological scales were generated from the individual items by simple summation to produce total scores. These scales were analysed further by linear modelling to determine the variance of the total scores across the demographics (i.e., sex and education), regularity of attendance and pandemic delay in dental attending. These comparisons can be summarised by referring to the statistically significant results only.

Two main approaches were applied to assess the psychometric qualities of both these scales (namely, the CAS and the CCAS, see Supplementary File). The data file was split randomly into two using a random number generator in STATA. All odd values of the identification number comprised one sub-sample and even numbers comprised the second sub-sample. The first analytical approach on the first sub-sample (*N* = 239) was to apply Horn’s Parallel factor analysis to validate that each scale possessed a unidimensional structure (Supplementary Figure S1). The second analytical approach was to conduct confirmatory factor analysis on the two scales simultaneously (using the second sub-sample of data, *N* = 264) so that the nine items were included in a two-factor model. Each scale was described as a latent variable with indicators defined solely by its own items. The results indicated reasonable fit in 12 iterations for these two scales when four error variances, determined by a table of modification indices, were introduced (Supplementary Table S1).

The procedure *Structural Equation Modelling* was applied and consisted of two stages. First, confirmatory factor analysis was conducted on each separate scale to determine unidimensionality and ascertain misspecification for each of the latent variables (e.g., treatment dental anxiety). Second, the structural model was tested with the combined measurement model and path analysis calculated simultaneously. Maximum likelihood estimation was utilised. Modification indices were inspected as well as close attention to the number of iterative steps required for convergence. Any Heywood values denoting excessive strains in the model specification were noted. Conventional fit criteria were applied including the Comparative Fit Index (CFI), Root Mean Square Error of Approximation (RMSEA) and Standardized Root Mean Square Residual (SRMR). Those modification indices that were very high were targets for enabling correlations between error terms in the measurement model. Further support from theoretical grounds was required prior to relaxing the constraint of independent errors. Each structural path was closely reasoned for retention or removal from the hypothesised model. That is, if the path exhibited poor covariation between the latent variables in question, then a repeat model was run excluding the non-significant path. Hence, a parsimonious approach was applied.

### Ethical consideration

The study was fully adhered to the Helsinki Declaration. We have received the ethical approval from the Ethics Committee of the School and Hospital of Stomatology, Zhejiang University of Chinese Medicine (reference number 02/202209/01). All the patient participants were fully informed of the research project and sought for a written consent before their participation.

## Results

The sample (*n* = 503, mean age = 37.7 years, SD = 12.0) consisted of a greater proportion (63%) of women respondents than men (37%), and with a large majority who indicated at least a level of education classified at a Higher Education category (Table 1). Only 18% reported only secondary or primary education attainment. A fifth of the sample approximately (21%) claimed regular attendance at the dentist and just over a third (35%) indicated that they had delayed their dental attendance due to the pandemic.

**Table 1:** Frequencies and means (SEs) for psychological scales across demographics, dental attendance and COVID-19 visit delay variables among a sample of Chinese dental patients (*n* = 503).

		%	(*N*)	CAS		CCAS		MDAS	
				Mean	(SE)	Mean	(SE)	Mean	(SE)
Variable									
Sex	Female	63	(315)	5.75	(0.10)	6.72	(0.14)*	8.64	(0.19)
	Male	37	(188)	5.61	(0.13)	6.16	(0.19)	8.50	(.025)
Education	Higher	82	(413)	5.59	(1.72)^a^	6.46	(0.13)^b^	8.45	(0.17)d
	Secondary	13	(63)	6.00	(2.22)	6.37	(0.32)^c^	8.84	(0.42)
	Primary	5	(27)	6.67	(1.83)^a^	7.66	(0.49)^b,c^	10.07	(0.65)d
Dental attendance	Regular	21	(104)	5.97	(0.18)	6.09	(2.40)	9.09	(0.33)
	Treatment	79	(399)	5.63	(0.09)	6.62	(2.61)	8.46	(0.17)
Delay attending	Yes	35	(177)	5.87	(0.14)	6.84	(0.19)*	8.83	(0.25)
due to COVID-19	No	64	(326)	5.60	(0.10)	6.33	(0.14)	8.45	(0.19)

Multiple comparison tests for the education variables (3 categories). Differences indicated by identical suffix letters.

Taking each scale in turn as presented in Table 1 the Covid Anxiety Scale (CAS) scores were clearly raised in those participants who had received only primary education compared with higher level education (*p *< .05). The Clinical care Covid Anxiety Scale (CCAS) scores were raised in women compared to men (*p *< .05) and in those who stated that they had delayed their dental appointment due to concerns over the pandemic (*p *< .05). Participants with primary education expressed greater anxiety about the clinical components of Covid than the other participants who had experienced greater levels of education. The variability of dental anxiety assessed by the MDAS was explained especially by the difference between respondents who had received only primary education compared with higher education. Consistent with the other two scales assessing various aspects of anxiety about issues associated with the pandemic those with primary education were on average more anxious than counterparts who had received higher education (*p *< .05).

**Figure 2:**
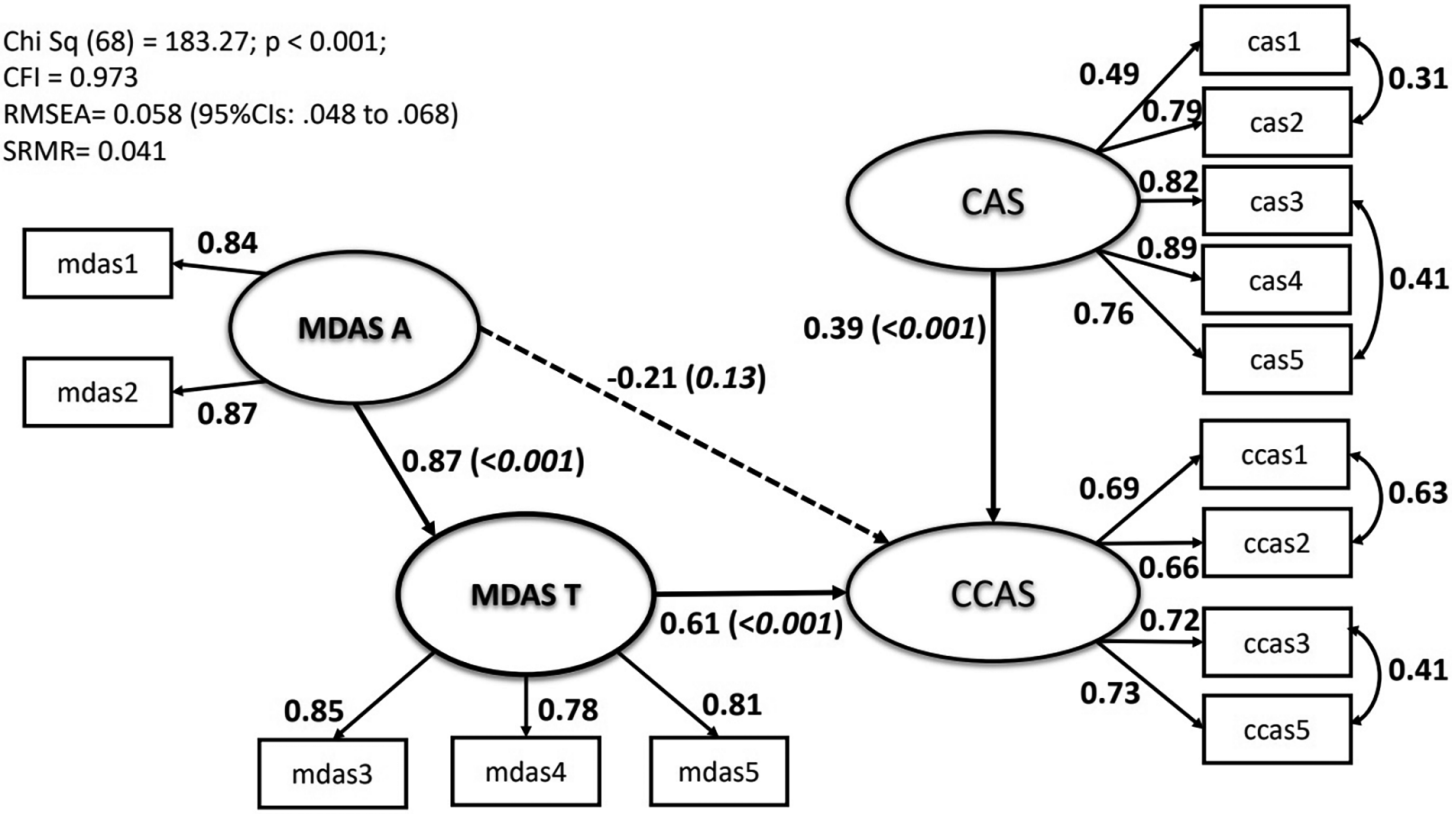
Resultant model tested showing path coefficients, item loadings onto latent variables and specified correlated errors. Direct effects: solid arrows; Indirect effect: broken line. Standardised coefficients presented. Residual errors and covariance between MDAS-A and CAS not shown to simplify diagrammatic representation. Fit statistics summarised in box, see text for full description.

A structural equation model (Figure 2) was constructed to test the ability of dental anxiety and the general Covid anxieties that respondents expressed to explain their clinical concerns about Covid when attending the dentist. The model was specified as a series of latent variables which possess beneficial qualities for the researcher including an explicit exposition of the relationships between the variables entered, a series of indices that reflect the degree of fit of the specified model and in addition a system (of modification indices) for alerting the researcher if changes to the specified model statistically might be considered. The dental anxiety scale (MDAS) was divided into its anticipatory and treatment components and expressed as separate latent variables MDAS_A and MDAS_T respectively (12). The anticipatory component of dental anxiety (MDAS_A) was considered to have some association to clinical Covid anxiety (CCAS) through its expression on treatment dental anxiety (MDAS_T). The model was expressed so that the anticipatory component was allowed to have a direct influence on clinical Covid anxiety. Similarly, general anxiety about Covid (CAS) was considered to have a direct effect on the clinical aspects of Covid anxiety (CCAS). The model was run and took 12 iterations to complete convergence. The error covariances that were present in the confirmatory factor analysis for the two Covid scales were introduced into this model and were shown to have strong effects in this structural model. The final model potentially had other constraints that might have been relaxed although theoretically they were not easily justified. So the final model was accepted as appearing in Figure 2. The fit statistics were acceptable [Chi-square = 183.27, df = 68, *p* < .001; CFI = 0.973; RMSEA = 0.058 (95%CIs: 0.048–0.068)] and the Standardised Root Mean Square Residuals (SRMR) index was 0.041. All standardised parameters between the items and latent variables were 0.49 or above. Only three of the 14 parameters were less than 0.7. The structural relationships between the dental anxiety latent variables and clinical Covid anxiety were strong and showed that anticipatory elements of dental anxiety fed anxieties of various common treatments known to occur for many in dental restorative and surgical specialities. No direct effect of anticipatory dental anxiety was shown to influence clinical concerns about covid (CCAS) (*p* = 0.13). The correlation between the anticipatory dental anxiety and general covid anxiety was 0.34 (not shown in Figure 2). Hence, the strength of associations in the pathways of the two different types of dental anxiety explaining clinical Covid anxiety are independent of general Covid anxiety, and likewise when focusing on general Covid anxiety to explain clinical concerns of Covid.

## Discussion

The present study aims to (i) identify the psychometric properties of the two COVID-19 scales (i.e., CAS and CCAS) and (ii) to assess the associations between the COVID-19 Anxiety Scale (CAS), dental anxiety (MDAS) and the clinical care COVID-19 Anxiety (CCAS) based on dental patients’ responses. Our study has shown good validity of the Clinical COVID-19 Anxiety Scale in the confirmatory factor analysis. The theoretical model we proposed, according to the Transactional Model of Stress and Coping, connects patient dental anxiety and COVID-19 anxiety to better understand patient’s concerns of contracting coronavirus from visiting a dental service. The findings show a good fit for our proposed theoretical model.

In terms of the bivariate analyses between anxiety scales (i.e., CAS, CCAS and MDAS), patient demographics, regularity of dental attendance and delay of dental attendance due to the pandemic, we found patients with primary education were more likely to report higher scores of CAS and CCAS. This is in line with results from a recent study in general Iranian population that respondents with limited education tended to report higher COVID-19 related anxiety score (14). In addition, our study shows women compared with men are also more likely to report higher CCAS scores. This has been echoed in other studies that women seemed more anxious about COVID-19 than men (6, 15). A possible explanation is that women might be more vigilant of the risk of coronavirus and are more likely to express their anxious feeling compared with men. Higher COVID-19 related anxiety scores from our study are also seen in those who claimed delay in dental attendance due to concerns over the pandemic. This is evident as people who are more concerned and anxious about COVID-19 would intend to avoid using dental services during the pandemic. Similar result has been found from a recent study that about one third of those claimed not to seek dental services are due to fear of infection from COVID-19 (6).

As stated previously, the Transactional Model of Stress and Coping provides a good theoretical framework to explain our proposed model (7). In our study, a patient who needs to visit a dental service for their non-COVID19 conditions may experience anxiety about the possibility of contracting corona virus, the safety of the dental hospital environment, the quality of care they will receive, and the impact of their condition on their life. Patients’ anxiety is influenced by their appraisal of the situation (primary appraisal, i.e., dental anxiety and general anxiety about COVID-19 pandemic) and their perceived ability to cope with it (secondary appraisal, i.e., their perceived anxiety of receiving dental treatment and risks of contracting COVID-19 infection from their dental visit). The two distinct paths identified from our findings have supported the theoretical basis and pinpointed the complex factors that contributed to patient development of anxiety associated with visiting dental facilities during the pandemic. It also may suggest that the CCAS is a more promising instrument to measure patient anxiety about receiving dental care due to these complex factors involving patient’s own dental anxiety and an infectious disease pandemic as another anxiety provoking factor.

There are certain limitations of the present study. First, we recruited a patient sample from a polyclinic of a dental hospital in East China. Moreover, the sample contained a larger proportion of women compared with men. This will restrict the representation of the sample and the generalisability of the study findings. Second, all the anxiety scales are based on participants’ self-reported responses that they may either provide more socially acceptable answers or not report their anxieties accurately. Third, patient anxiety is complex, which can be influenced by many other factors such as patient previous experience of COVID-19 exposure, family history of COVID-19 exposure as well as their socioeconomic factors. For pragmatic reasons due to resources and to limit questionnaire burden on the respondent, we could not explore these factors in our study. We acknowledge that there might be an interplay between these additional factors and the variables that we explored. Nevertheless, to our knowledge, this is the first study to explore psychological factors including both dental anxiety and pandemic related fears that responsible for patient fear for attending for dental treatment. Moreover, the large sample size of the present study can allow us to investigate the associations between the three psychological measurements more accurately.

Our findings can help researchers and dental professionals understand the complex factors that may contribute to patient anxiety and fears about visiting dental health service during pandemics. By identifying these factors and developing strategies to address them, dental professionals can help alleviate patient anxiety and improve their care experience to promote positive health outcomes. This can be realised through effective infection control measures and communication with patients, as well as providing access to tele-dentistry and mobile dental facilities.

## Conclusion

Our study has shown that the Clinical care COVID-19 Anxiety Scale has demonstrated satisfactory psychometric properties to test people’s fear of being infected by COVID-19 while using dental facilities in a sample of Chinese patients attending in a dental hospital in East China. It also shows both the dental anxiety and the general anxiety about the pandemic have strong associations to patients’ fear of contracting corona virus from their dental attendance and not receiving appropriate dental care. Our study has practical implications to help healthcare providers better understand how environmental stressors such as the pandemic related fears along with patient’s mental health conditions (e.g., dental anxiety) influence patients’ overall concerns on infection risks and appropriate dental treatments they will receive during the pandemic.

## Data Availability

The raw data supporting the conclusions of this article will be made available by the authors, without undue reservation.
