# Supplementary material for: Does fear of infection affect people’s dental attendance during COVID-19? A Chinese example to examine the association between COVID anxiety and dental anxiety
Source: Front Oral Health. 2023 Oct 9;4:1236387. doi: 10.3389/froh.2023.1236387 (PMC10591092; doi:10.3389/froh.2023.1236387)
Supplement: Supplementary file 1 [file Table1.docx]

Table S1 Confirmatory Factor Analysis, standardized solution, factor loadings, residual variances, covariances between imposed correlated errors and correlation of the two latent variables

The internal consistency of these two scales were as follows:

|  | Cronbach alphas | 95%CIs | |
| --- | --- | --- | --- |
|  |  | low | high |
| CAS | 0.878 | 0.863 | 0.892 |
| CCAS | 0.849 | 0.830 | 0.868 |
