# Supplementary material for: Does fear of infection affect people’s dental attendance during COVID-19? A Chinese example to examine the association between COVID anxiety and dental anxiety
Source: Front Oral Health. 2023 Oct 9;4:1236387. doi: 10.3389/froh.2023.1236387 (PMC10591092; doi:10.3389/froh.2023.1236387)
Supplement: Supplementary file 2 [file Presentation1.pdf]

Supplementary File: CAS and CCAS Psychometrics

This brief file provides some psychometric background information on the two psychological rating scales. Specifically these comprised of two scales, as follows:

1. Covid Anxiety Scale (CAS). This scale consists of 5 items and has already been published by Sherman Lee in the journal of Death Studies, entitled Covid Anxiety Scale: A brief mental health screener for COVID-19 related anxiety (2020) Volume 44, no.7, 393-401. However the scale has not reported detailed psychometrics for a Chinese translation (Madarin).
2. Clinical Care and Covid Anxiety Scale (CCAS). This scale was constructed by Humphris and Yuan as a simple set of 4 items which focused on the patients' ratings on when visiting oral health services on their anxiety about: timeliness / effectiveness of treatment and susceptibility to the COVID virus and their survival.

Two main approaches were applied to assess the psychometric qualities of both these scales. Data were derived from the cross-sectional study presented in this paper. The data file was split randomly into two using a random number generator in STATA. All odd values of the identification number comprised one sub-sample and even numbers comprised the second sub-sample.

The first analytical approach on the first sub-sample (N = 239) was to apply Horn's Parallel factor analysis to validate that each scale possessed a unidimensional structure. That is, the scales when analysed separately determine whether the items would best fit a single dimension. A common but flawed methodology is to use what has been termed the Kaiser criteria of unity, which refers to adopting, what may be regarded, as an artificial eigenvalue of one. In other words, dimensions within the measure that possess eigenvalues greater that one are allowed to assume factorial status. Horn's framework derives a randomly prepared data file sampled from the raw data itself and then factor analyses this data file. Then the actual data file is subjected to the same routine and the criteria for acceptance of distinct factors is based upon the random data structure calculation as opposed to the artificial unity indicated by the Kaiser solution. Adjustment to avoid bias is included for the number of items that are candidates for the data reduction procedure. The results of the two separate analyses (one for each scale: CAS and CCAS) are presented diagrammatically, demonstrating clearly that each scale possess a unidimensional structure (Figure S1, panels A and B). Panel A is presented on the Left and Panel B is shown on the Right hand side.

The second analytical approach was to conduct confirmatory factor analysis on the two scales simultaneously (using the second sub-sample of data, N = 264) so that the nine items were included in a two factor model. Each scale was described as a latent variable with indicators defined solely by its own items. No cross-over from latent variables to the other scale's indicators was allowed. The standardised solution of factorial loadings are presented in the table below and the statistical significance of each factor loading was determined. In order, to assess the fit, the conventional indices of CFI, RMSEA and SRMR were inspected, in addition to the chi square value. The later is not easy to interpret as sample size tends to distort the significance level so that fit would unlikely be recognised. The results indicated reasonable fit in 12 iterations for these two scales when four error variances, determined by a table of modification indices, were introduced (Table S1). The common wording employed in some pairs of items in each of the scales appeared to be responsible for these distortions and were encouraged to remain in the model. The statistical fit indices were: CFI = 0.97; RMSEA = 0.086 (95%CI 0.086 , 0.110), SRMR = 0.051 showing a reasonable level of fit. The chi square with 23 degrees of freedom was 67.88 and significant at the .00001. There were no Heywood figures or extensive iterations. No cross loadings were indicated in the measurement model.
