# Supplementary figures and images for: Does fear of infection affect people’s dental attendance during COVID-19? A Chinese example to examine the association between COVID anxiety and dental anxiety
Source: Front Oral Health. 2023 Oct 9;4:1236387. doi: 10.3389/froh.2023.1236387 (PMC10591092; doi:10.3389/froh.2023.1236387)

Figure S1. The unidimensional structure of CAS and CCAS

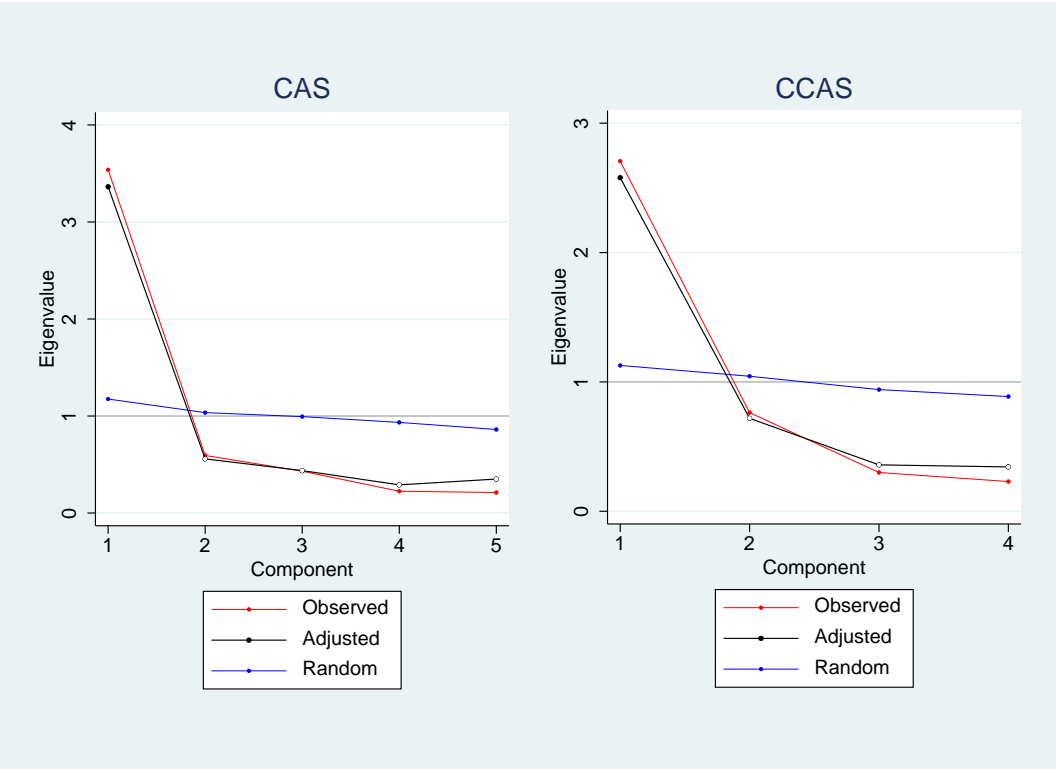

Supplement: Supplementary file 3 [file Image1.pdf]
